# Polymerized Albumin Receptor of Hepatitis B Virus for Evading the Reticuloendothelial System

**DOI:** 10.3390/ph14050408

**Published:** 2021-04-25

**Authors:** Kurumi Takagi, Masaharu Somiya, Joohee Jung, Masumi Iijima, Shun’ichi Kuroda

**Affiliations:** 1 Graduate School of Bioagricultural Sciences, Nagoya University, Nagoya 464-8601, Japan; kurumi.takagi@gmail.com (K.T.); mi206786@nodai.ac.jp (M.I.); 2 Department of Biomolecular Science and Reaction, The Institute of Scientific and Industrial Research, Osaka University, Osaka 567-0047, Japan; msomiya@sanken.osaka-u.ac.jp; 3 College of Pharmacy, Duksung Women’s University, Seoul 132-714, Korea; joohee@duksung.ac.kr; 4 Department of Nutritional Science and Food Safety, Faculty of Applied Biosciences, Tokyo University of Agriculture, Tokyo 156-8502, Japan

**Keywords:** albumin, bio-nanocapsule, hepatitis B virus, nanoparticle, polymerized human serum albumin receptor, reticuloendothelial system

## Abstract

Various strategies, such as optimization of surface chemistry, size, shape, and charge, have been undertaken to develop nanoparticles (NPs) as DDS (drug delivery system) nanocarriers for evading the reticuloendothelial system (RES) in vivo. We previously developed a hollow NP composed of hepatitis B virus (HBV) surface antigen L proteins and lipid bilayers, hereinafter referred to as bio-nanocapsule (BNC), as a nonviral DDS nanocarrier. Such a BNC harbors the HBV-derived human hepatic cell-specific infection mechanism, and intravenously injected BNCs by themselves were shown to avoid clearance by RES-rich organs and accumulate in target tissues. In this study, since the surface modification with albumins is known to prolong the circulation time of nanomedicines, we examined whether the polymerized albumin receptor (PAR) of BNCs contributes to RES evasion in mouse liver. Our results show that NPs conjugated with peptides possessing sufficient PAR activity were captured by Kupffer cells less efficiently in vitro and were able to circulate for a longer period of time in vivo. Comparing with polyethylene glycol, PAR peptides were shown to reduce the recognition by RES to equal content. Taken together, our results strongly suggest that the PAR domain of BNCs, as well as HBV, harbors an innate RES evasion mechanism. Therefore, the surface modification with PAR peptides could be an alternative strategy for improving the pharmacodynamics and pharmacokinetics of forthcoming nanomedicines.

## 1. Introduction

For several decades, nanoparticles (NPs) have been recognized as promising nanocarriers for delivering imaging and therapeutic agents, such as fluorophores, drugs, and genetic materials. With regard to the systemic administration of NPs, many studies have raised issues concerning their rapid clearance from the bloodstream via the interactions with the reticuloendothelial system (RES) [1]. Various phagocytes, including macrophages, monocytes, and dendritic cells, clustered in RES of liver, spleen, and lung are likely to capture circulating NPs immediately and hamper the delivery of NPs to specific tissues and organs. Phagocytosis of NPs is known to be regulated by two major mechanisms: opsonization-dependent and opsonization-independent mechanisms [2]. The former is triggered by the opsonization of the NP surface, and NPs that are modified with opsonins (e.g., antibodies, complements, and fibronectins) in the bloodstream are then recognized by either Fcγ receptors or complement receptor 3 on phagocytes and promptly excluded from the bloodstream [3]. The latter mechanism is triggered by negative charges of NPs and mediated by scavenger receptors [4].

Since the physicochemical properties (i.e., size, shape, charge, and surface chemistry) of NPs determine the recognition efficiency by RES, it is indispensable to optimize these properties of forthcoming drug delivery system (DDS) nanocarriers to reduce recognition by RES. The surface chemistry of NPs should be optimized to inhibit both the adsorption of opsonins and recognition by phagocytes in RES. Several studies have reported that modifications with hydrophilic polymers, such as polyethylene glycol (PEG) [5] and polysaccharide (dextran) [6], and membrane-derived components, for example, ganglioside GM1 [7] and heparin [6], were able to either increase steric hindrance or form a hydrated layer on the surface of NPs, thereby improving their pharmacodynamics and pharmacokinetics. However, unfortunately, the repetitive administration of PEGylated NPs was found to induce accelerated blood clearance by eliciting anti-PEG IgM [8]. Polysaccharides with certain configurations on NPs could also behave as complement activators [9], thereby inducing the capture by complement receptor 3. In addition, steric hindrance caused by these hydrophilic polymers may weaken the interaction of NPs with target cells and therefore repress the drug release from NPs [10,11,12]. To date, while effective strategies have been developed for each specific NP to evade RES, they have not been applicable to every NP. Therefore, an optimal strategy should be carefully chosen for each NP, according to the type of NPs and indications of NP-based nanomedicines, and, if necessary, several strategies should be combined to generate a synergetic effect. Altogether, it is a significant challenge to expand the number of choices for evading RES by establishing novel strategies for forthcoming DDS nanocarriers.

**Figure 1:**
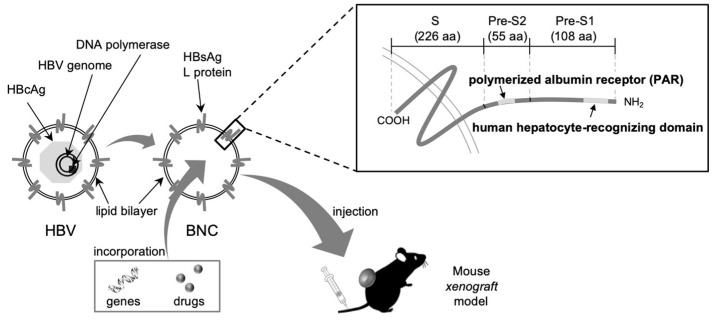
Structures of Bio-nanocapsule (BNC) and hepatitis B virus (HBV). HBV is composed of HBsAg embedded in lipid bilayer, HBcAg (HBV core antigen), DNA polymerase, and the HBV genome. Similarly, BNC is composed of HBsAg L proteins embedded in lipid bilayer. The L protein contains three domains, including the pre-S1 region containing a human hepatocyte-recognizing domain, the pre-S2 region with a polymerized albumin receptor (PAR) domain, and the S region with three transmembrane-spanning segments. The number of amino acids in each domain is indicated in parentheses. BNCs could be used for the in vivo pinpoint delivery of genes and drugs in mouse models via intravenous injection.

In 2003, we developed bio-nanocapsules (BNCs) as a nonviral DDS nanocarrier (Figure 1), which consists of hepatitis B virus (HBV) surface antigen (HBsAg) L proteins embedded in lipid bilayer [13] and therefore has an external appearance similar to that of HBV. BNC is a hollow capsule (about 100-nm) synthesized in *Saccharomyces cerevisiae* and is able to incorporate and deliver drugs and genes specifically to human hepatic tissues in vivo by utilizing a HBV-derived infection mechanism [14]. The HBsAg L protein contains the following three structural regions from the N terminus: pre-S1 region (108-amino-acid) including a human hepatocyte-recognizing domain indispensable for HBV infection [15,16,17]; pre-S2 region (55-amino-acid) containing a polymerized albumin receptor (PAR) with high affinity to polymerized human serum albumin (pHSA) [18]; and S region (226-amino-acid) necessary for self-assembly of HBsAg particles. When injected intravenously to a mouse harboring human normal liver tissues under its kidney skin, such BNCs were found to accumulate exclusively in the transplanted tissues without being delivered to any other tissues [19]. In addition, BNCs were also capable of delivering the green fluorescent protein (GFP) gene specifically to human hepatocellular carcinoma-derived tumors in a mouse x enograft model [20]. Importantly, the modification of LPs containing the anticancer drug doxorubicin with BNCs was further shown to prolong the half-life of LPs in the blood, and the BNC-LP complex was able to effectively suppress the progression of human hepatocellular carcinoma-derived tumors in the mouse xenograft model [21]. Taken together, these results have strongly suggested that BNC itself may possess a RES evasion mechanism, which would be derived from HBV.

In this study, we postulate that PAR in the pre-S2 region participates in the putative RES evasion mechanism by recruiting HSA on BNCs, since HSA-based NPs and HSA-conjugated molecules were shown to exhibit a long half-life in the blood [22,23,24]. Overall, our results demonstrate that PAR on BNCs indeed contributes to the evasion from RES and that the PAR-containing peptide endows the RES evasion activity to certain NPs.

## 2. Results and Discussion

### 2.1. In Vivo Biodistribution of BNC-LP Complexes

**Figure 2:**
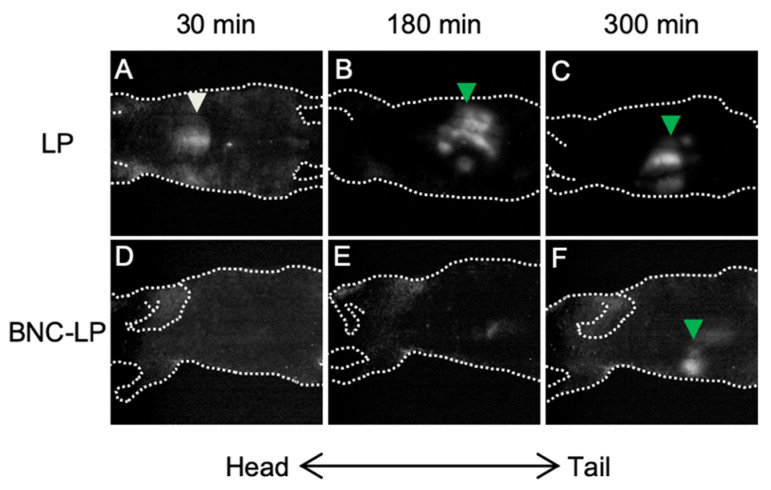
In vivo imaging analysis of LPs and BNC-LP complexes. Each mouse was injected intravenously with Rh-labeled LPs, either with or without 10 µg of BNCs. After 30, 180, and 300 min, Rh-derived fluorescence was detected from the ventral side of mice using an in vivo imaging system OV-100. The orientation (head and tail) of mice is shown by arrows. White and green arrowheads indicate liver and intestine, respectively.

Previously, it was demonstrated that intravenously injected BNCs accumulated exclusively in transplanted human hepatic tissues in mice [19,20] and that surface modifications of LPs with BNCs were able to prolong their half-life in the blood [25], suggesting that BNCs may harbor RES evasion activity. In this study, we prepared and injected Rh-labeled forms of LPs and BNC-LP complexes intravenously to mice and then compared their biodistribution by in vivo imaging (Figure 2). We observed that while LPs accumulated in livers after 30 min and excreted into the intestine (probably via biliary excretion) after 180 min (Figure 2B,C), the BNC-LP complexes circulated in the body without accumulating in specific organs for at least 180 min and were finally present in the intestine (Figure 2F). As the diameter and ζ-potential of both LPs and BNC-LP complexes were similar (about 150-nm and negatively charged; Table 1), these results prompted us to investigate the domain(s) responsible for the postulated RES evasion activity of BNCs.

### 2.2. Binding of BNCs to Various Albumins

**Figure 3:**
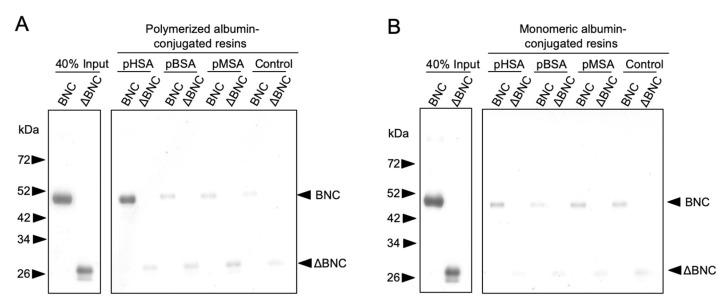
In vitro albumin-binding activity of BNCs. Polymerized (**A**) and monomeric (**B**) forms of human (HSA), bovine (BSA), or mouse (MSA) albumins that were conjugated to resins were incubated with either BNCs or ΔBNCs at 37 °C for 1 h, washed with PBS 4 times, subjected to 12% SDS-PAGE, followed by silver staining. The amounts of precipitated BNCs and ΔBNCs were determined by densitometry. Forty percent input, the loading control. ΔBNCs, PAR-deleted BNCs.

The pre-S2 region of HBV is known to function as PAR and exhibit strong affinity to polymerized human serum albumin (pHSA). Such pHSA recruitment was reported to improve the interaction of HBV with hepatocytes and thereby enhance HBV infectivity [26]. Notably, HSA is the most abundant plasma protein (35–50 g/L of human serum) with an average half-life of 19 days [24], and a substantial portion is polymerized by oxidative stress in the bloodstream [27]. The serum concentration of polymerized HSA in healthy human was estimated to 4 mg/L [28]. It has been demonstrated that HSA-based NPs and HSA-conjugated molecules exhibited long circulation half-lives in the blood [22,23,24]. Therefore, in this study we investigated whether the recruitment of endogenous albumins onto HBV, as well as BNCs, by PAR activity contributes to their RES evasion mechanism. After conjugation with Sepharose beads, each albumin (human, bovine, or mouse) in either a monomeric or polymerized form was incubated with BNCs or ΔBNCs (trypsinized BNCs), washed with PBS thoroughly, subjected to SDS-PAGE, followed by silver staining (Figure 3). Our results show that among all polymerized and monomeric albumins tested, only pHSA interacted with BNCs efficiently. As expected, ΔBNCs did not show affinity to any albumins examined, confirming that the pre-S region (most likely the pre-S2 region) acts as PAR and shows high affinity to pHSA [18]. Next, following the adsorption of BNCs onto the QCM sensor chip, the avidity of BNCs to each albumin was determined (Table 2). We found that BNCs showed strong and weak avidity to pHSA and pMSA, respectively, but did not bind to monomeric albumins (HSA or MSA). The finding of a substantial avidity of BNCs to pMSA strongly suggests that intravenously injected BNCs may recruit endogenous pMSA onto their surface, thereby contributing to their RES evasion activity.

### 2.3. Affinity of BNC-Derived Peptides to pHSA

**Figure 4:**
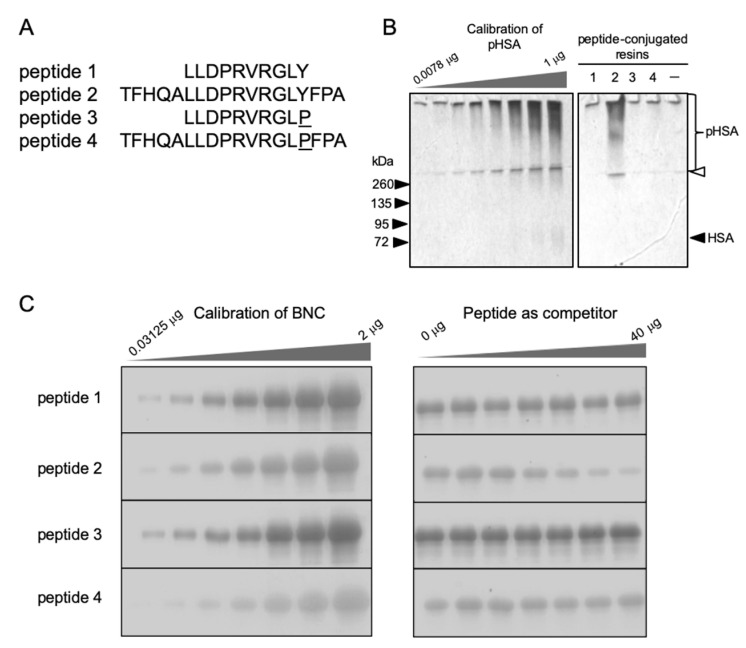
In vitro pHSA-binding activity of BNC-derived peptides. (**A**) Synthetic peptides containing the putative PAR region. Peptide 1, Leu-12 to Tyr-21 of the pre-S2 region; peptide 2, Thr-7 to Ala-24 of the pre-S2 region; peptide 3, peptide 1 containing a mutation of Tyr-21 to Pro-21 (underlined); and peptide 4, peptide 2 containing a mutation of Tyr-21 to Pro-21 (underlined). (**B**) Pull-down assays with peptide-conjugated resins (right panel). Left panel, calibration of pHSA. Resins were incubated with pHSA at 37 °C for 1 h, washed with PBS 4 times, subjected to 12% SDS-PAGE, followed by silver staining. The border between stacking and separating gels is indicated by a white arrowhead. (**C**) Competition assays using pHSA-conjugated resins (right panels). Left panels, calibration of BNCs (0.03125, 0.0625, 0.125, 0.25, 0.5, 1, and 2 μg as protein). Resins (containing 10 μg pHSA) were incubated with BNCs (20 μg) in the presence of specific peptides (0, 1.25, 2.5, 5, 10, 20, and 40 μg) at 37 °C for 1 h, washed with PBS 4 times, subjected to 12% SDS-PAGE, followed by silver staining. The amounts of precipitated BNCs were determined by densitometry.

Imai et al. [30] was the first to demonstrate that HBV and HBsAg particles contain the PAR protein. A HBV mutant lacking PAR activity has been shown to lose its infectivity in chimpanzees, suggesting that PAR is involved in the HBV infection machinery [26]. Previously, our group delineated that the region responsible for PAR activity is between residues Leu-12 and Tyr-21 of the pre-S2 region [31]. Here, a peptide containing the putative PAR region (Leu-12 to Tyr-21; peptide 1), as well as a peptide encompassing the putative PAR region (Thr-7 to Ala-24; peptide 2), were synthesized (Figure 4A) and examined for their PAR activity by pull-down assays. As shown in Figure 4B, peptide 2-conjugated resins showed stronger affinity to pHSA than peptide 1-conjugated resins, suggesting that the flanking regions of the putative PAR region are necessary for sufficient PAR activity. Next, we compared the PAR activity of these two peptides in competition assays using pHSA-conjugated resins (Figure 4C). We found that only peptide 2 successfully repressed the interaction between BNCs and pHSA in a dose-dependent manner. On the other hand, when residue Tyr-21 of peptides 1 and 2 was replaced with Pro to generate peptides 3 and 4, respectively, peptide-conjugated resins were not able to interact with pHSA (Figure 4B) or to interfere with the interaction between BNCs and pHSA (Figure 4C). Since the PAR region was postulated to be located between two putative helixes (from Met-1 to Leu-13 and from Leu-20 to Phe-46) [31], the Pro-21 mutation might work as a breaker against the second helix and thereby affect the PAR function.

### 2.4. Effect of BNC-Derived Peptides on Phagocytosis by Kupffer Cells

Since the liver is a major RES-rich organ in the body, DDS nanocarriers need to evade phagocytosis by Kupffer cells (i.e., liver-specific phagocytes). As shown in Figure 2, intravenously injected BNC-LP complexes, unlike LPs, did not accumulate in mouse liver, strongly suggesting that BNCs were poorly captured by Kupffer cells. To examine the effect of surface modifications of LPs with albumins or peptide 2 on the uptake by phagocytes, fluorophore (NYO)-labeled polystyrene microspheres, whose size is comparable to that of BNCs, were modified with various molecules (Table 3) by incubation in 50% (*v*/*v*) mouse serum at 37 °C for 30 min.

**Figure 5:**
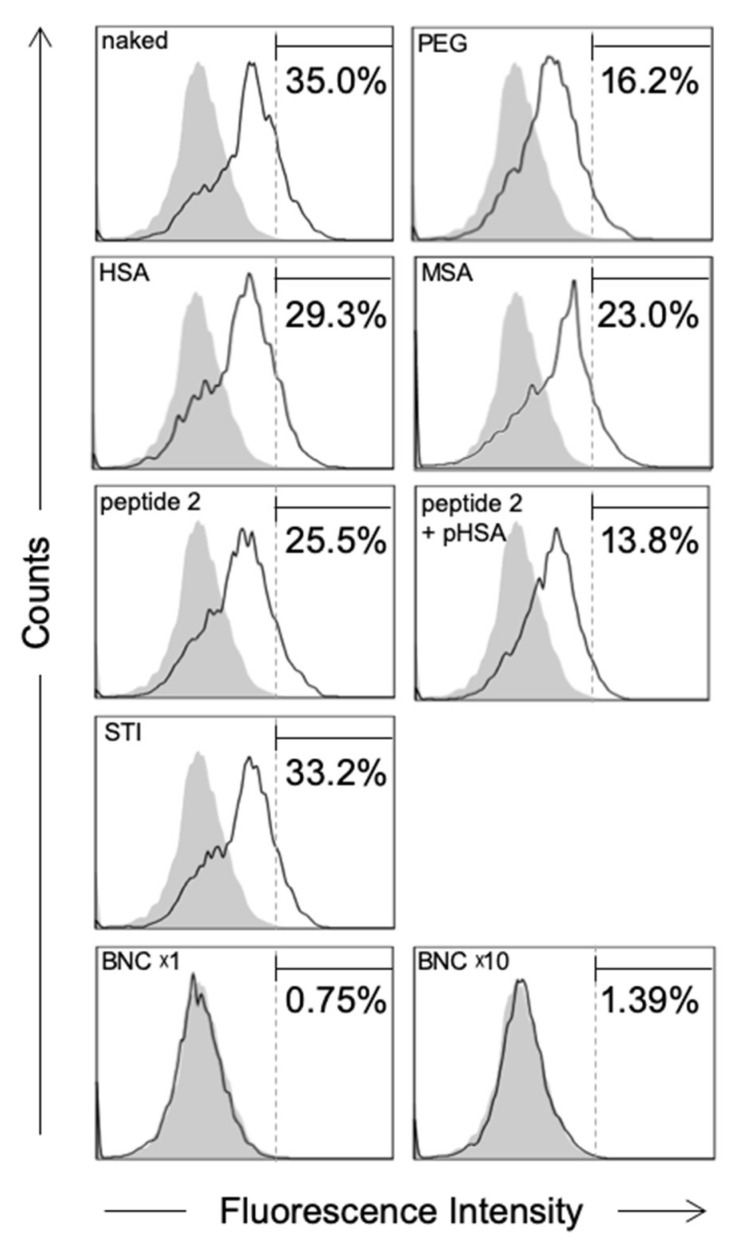
In vitro uptake of fluorophore-labeled microspheres and BNCs by Kupffer cells. After opsonization of Cy3-labeled BNCs and NYO-labeled microspheres (naked, PEG-modified, HSA-modified, MSA-modified, peptide 2-modified with/without pHSA treatment, or STI-modified), about 5 × 10^4^ Kupffer cells were incubated with the NYO-labeled microspheres (about 5 × 10^8^ particles) or Cy3-labeled BNCs (about 5 × 10^8^ or 5 × 10^9^ particles) at 37 °C for 30 min and subjected to the FACS analysis. The fractions of Kupffer cells were predefined by the forward scatter/side scatter dot plots derived from CD11b^+^ cells. Distributions of microspheres and BNCs in Kupffer cells were indicated as open histogram. Controls (untreated Kupffer cells) were indicated as shaded histograms. The percentages (%) of NYO-labeled or Cy3-labeled Kupffer cells were presented as numbers.

Next, about 5 × 10^4^ primary Kupffer cells were incubated with these microspheres (about 5 × 10^8^ particles) at 37 °C for 30 min and then subjected to the FACS analysis. Our results show that while naked microspheres were incorporated by about 35.0% of total Kupffer cells, HSA- and MSA-modified microspheres were incorporated by about 29.3% and 23.0% of total cells, respectively (Figure 5). In addition, PEG-modified microspheres (positive control) were captured by about 16.2% of total cells [5]. These findings supported that the modification with albumins confers sufficient RES evasion activity to microspheres [22]. In contrast, under the same conditions, soybean trypsin inhibitor (STI)-modified microspheres (negative control) were not able to evade the uptake by Kupffer cells (about 33.2% of total cells). Notably, peptide 2-modified microspheres were also found to evade the capture by Kupffer cells (about 25.5% of total cells) at a comparable level to both HSA- and MSA-modified microspheres. Interestingly, the RES evasion efficacy of peptide 2 was further enhanced by preincubation with pHSA (about 13.8% of total cells). In agreement with the report by Ogawara et al. [22] that the surface modification of NPs with albumins suppressed the adsorption of opsonins, our results strongly suggest that the surface modification of NPs with PAR-containing peptides is effective for preventing the uptake by Kupffer cells, which in turn recruits serum albumins onto the surface of NPs and prevents them from opsonization. It has been known that protein corona formation on the surface of NPs may play a significant role on the pharmacokinetics [32,33,34]. Our results suggested that surface modification of NPs with PAR-containing peptide may affect the protein corona formation; however, further investigation is needed. Moreover, we found that BNCs (approximately the same amounts of particles) were hardly captured by Kupffer cells (about 0.75% of total cells), even when 10-fold more BNCs were used (about 1.39% of total cells), indicating that BNCs possess more efficient RES evasion activity than PAR peptide-modified microspheres. Significant differences in RES evasion activity between these two types of NPs might be attributed to either the oriented immobilization of pre-S peptides on BNCs or unidentified endogenous RES evasion machineries of HBV.

### 2.5. Effect of BNC-Derived Peptides on Circulation Time of NPs

**Figure 6:**
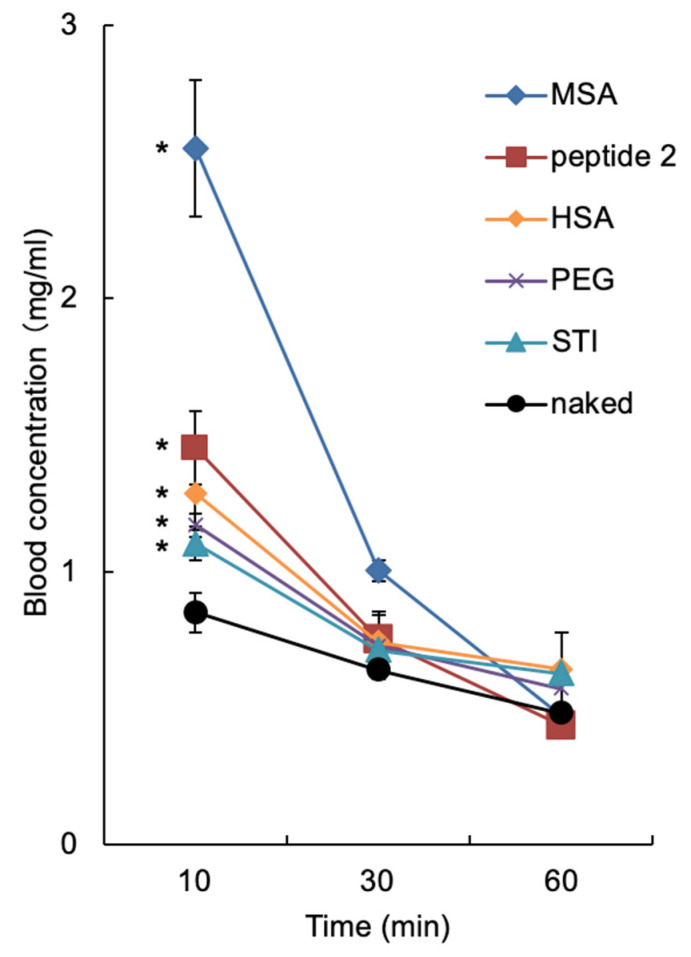
Blood concentration–time curves of NYO-labeled microspheres. At 10 min, 30 min, and 60 min after intravenous injection with NYO-labeled microspheres (100 μg/mouse), blood samples were collected and processed as described in Materials and Methods. The concentrations of fluorophores were determined by a fluorescence microplate reader (n = 5; mean ± SEM; *t*-test, * *p* < 0.05).

Based on the results described above, the modification with albumins or peptide 2 was expected to prolong the serum circulation time of the microspheres by reducing the uptake by RES, since the recognition by Kupffer cells was decreased. In this study, mice were intravenously injected with the microspheres used in Figure 5 (100 μg as microspheres), and fluorophores contained in microspheres were extracted from whole blood with chloroform, and the fluorescence intensity was measured. As shown in Figure 6, the surface modification with MSA was found to prolong the serum circulation time more extensively than other modifications. Compared to naked microspheres, all other molecules were capable of effectively extending the serum circulation time of microspheres in the following order: peptide 2, HSA, PEG (positive control [5]), and STI (negative control). In the light of these results, the surface modification of NPs with albumins or peptide 2 was an effective strategy for evading RES in vivo.

### 2.6. Effect of BNC-Derived Peptides on Hepatotropic Properties of NPs

**Figure 7:**
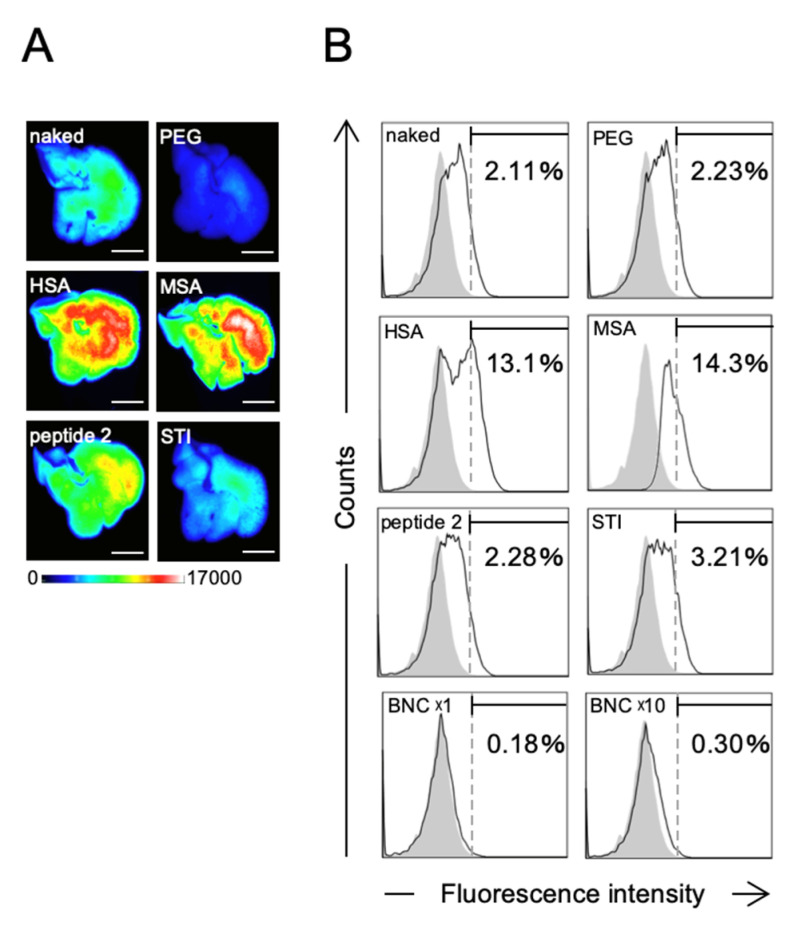
Evaluation of hepatotropic properties of microspheres and BNCs. (**A**) In vivo uptake of fluorophore-labeled microspheres by mouse livers. About 100 μg of NYO-labeled microspheres (naked, PEG-modified, HSA-modified, MSA-modified, peptide 2-modified, or STI-modified) were administrated intravenously into each mouse. After 10 min, livers were isolated and subjected to the in vivo imaging analysis. The color bar shows NYO-derived fluorescence intensity. Bar, 1 cm. (**B**) After opsonization of Cy3-labeled BNCs and NYO-labeled microspheres (naked, PEG-modified, HSA-modified, MSA-modified, peptide 2-modified, or STI-modified), about 5 × 10^4^ mouse primary hepatocytes were incubated with NYO-labeled microspheres (about 1 × 10^9^ particles) or Cy3-labeled BNCs (about 1 × 10^9^ or 1 × 10^10^ particles) at 37 °C for 30 min and then subjected to the FACS analysis. Distributions of microspheres and BNCs in hepatocytes were indicated as open histograms. Controls (untreated hepatocytes) were indicated as shaded histograms. The percentages (%) of NYO-labeled or Cy3-labeled hepatocytes were presented as numbers.

From the mice receiving intravenous injection of microspheres described above, livers were isolated after 10 min and subjected to the in vivo imaging analysis. Unexpectedly, these results show that both MSA- and HSA-modified microspheres were remarkably accumulated in livers, compared to naked, PEG-modified, peptide 2-modified, or STI-modified microspheres (Figure 7A), inconsistent with the observation that MSA- and HSA-modified microspheres were able to escape from the uptake by Kupffer cells and then evade RES (see Section 2.4). To resolve this discrepancy, we examined whether hepatocytes can efficiently incorporate these albumin-modified microspheres. Mouse primary hepatocytes (about 5 × 10^4^ cells) were incubated with opsonized microspheres (about 1 × 10^9^ particles) in PBS at 37 °C for 30 min and then subjected to the FACS analysis. We found that MSA- and HSA-modified microspheres were efficiently captured by hepatocytes, compared to other microspheres (naked, PEG-modified, peptide 2-modified, or STI-modified microspheres) (Figure 7B). Meanwhile, it has been postulated that the liver polymerized-albumin receptor on the surface of hepatocytes plays a pivotal role in the uptake of serum albumins by livers [35] and that the neonatal Fc receptor (FcRn) localized in the endosome of hepatocytes is involved in the prolongation of the serum half-life of albumins [36] and albumin-modified molecules [37]. Specifically, FcRn interacts with the albumin moiety of these molecules in late endosomes, thereby diverting them from lysosomal degradation and returning them to the extracellular compartment [38]. Collectively, we suggest that MSA- and HSA-modified microspheres were captured by hepatocytes, but not Kupffer cells, via liver polymerized-albumin receptor and were promptly excreted from hepatocytes via FcRn. Such a recycling pathway mediated by FcRn might make livers serve as a reservoir for both microspheres, thereby retaining their high concentrations in the blood. While peptide 2-modified microspheres were found to escape from the uptake by Kupffer cells by displaying polymerized albumins (see 3.4), they were not incorporated efficiently by hepatocytes. These results strongly suggest that polymerized HSA on the peptide 2-modified microspheres was not efficiently captured by the liver polymerized-albumin receptor of hepatocytes. Therefore, compared to the surface modification with the monomer form of albumins, the modification with peptide 2 would be more useful for improving the pharmacodynamics and pharmacokinetics of forthcoming nanomedicines.

## 3. Materials and Methods

### 3.1. Bio-Nanocapsules (BNC), Liposomes (LPs), and BNC-LP Complexes

BNCs overexpressed in *Saccharomyces cerevisiae* AH22R cells carrying the BNC-expression plasmid, pGLDLIIP39-RcT, were purified as described previously [39]. Protein concentrations were determined using a BCA protein assay kit (Pierce, Rockford, IL, USA) with bovine serum albumin (BSA; Wako Pure Chemical Industries, Osaka, Japan) as a control protein. Dipalmitoylphosphatidylcholine (DPPC; NOF, Tokyo, Japan), dipalmitoylphosphatidylethanolamine (DPPE; NOF), dipalmitoylphosphatidylglycerol sodium (DPPG-Na; NOF), and cholesterol (Chol; Nakalai Tesque, Kyoto, Japan) were dissolved in a chloroform/methanol (4:1, *v*/*v*) mixture in a round-bottom flask (DPPC: DPPE: DPPG-Na: Chol = 15:15:30:40, mol/mol) and allowed to evaporate at room temperature in a rotary evaporator to produce a thin hemispherical lipid film. To produce about 100-nm anionic LPs, the film was hydrated in distilled water and sonicated for 6 min at room temperature using an Astrason ultrasonic disruptor (Misonix, Farmingdale, NY, USA). Average diameters and ζ-potentials of LPs were measured at 25 °C in phosphate-buffered saline (PBS; 140 mM NaCl, 2.7 mM KCl, 10 mM Na_2_HPO_4_, 1.8 mM KH_2_PO_4_) by a dynamic light scattering (DLS) model Zetasizer Nano ZS (Malvern Instruments, Worcestershire, UK). To prepare fluorescent LPs, lissamine rhodamine B-1,2-dihexadecanoyl-sn-glycero-3-phosphoethanolamine (Rh-DHPE; Invitrogen, Carlsbad, CA) was added to the preparation of LPs at a final concentration of 5% (mol/mol). A mixture of Rh-labeled LPs and BNCs was suspended in Britton–Robinson buffer (pH 4.0; 32 mM H_3_PO_4_, 32 mM CH_3_COOH, 32 mM H_3_BO_3_, 40 mM NaOH) at a LP/BNC ratio of 2:1 (*w*/*w*), incubated at 37 °C for 30 min, and subjected to isopycnic ultracentrifugation (P40ST rotor; Hitachi, Tokyo, Japan) through a CsCl gradient (10–40% (*w*/*v*)) at 24,000 rpm for 16 h at room temperature. Fractions containing BNC-LP complexes were combined and dialyzed against PBS overnight at 4 °C.

### 3.2. In Vivo Imaging

Mice were handled according to the guidelines of Graduate School of Bioagricultural Sciences, Nagoya University, Japan. Animal experiments described in this study were approved by the animal experiment committee in Nagoya University (approved number 2010031805). Each female Balb/c mouse (6 weeks old, CREA Japan, Tokyo, Japan) was injected intravenously with 50 µL of PBS containing 10 µg (as protein) of BNC-LP complexes. After 30, 180, and 300 min, Rh-derived whole-body fluorescent signals were measured by an in vivo imaging system OV-100 (Olympus, Tokyo, Japan) equipped with a xenon lamp and emission filters (from 535–555 nm) and analyzed using WASABI software (Hamamatsu Photonics, Shizuoka, Japan).

### 3.3. Pull-Down Assays with Albumin-Conjugated Resins

Human and mouse serum albumin (HSA and MSA, respectively; Sigma Aldrich, Atlanta, GA, USA) and BSA were dissolved in PBS (final concentration, 2.5% (*w*/*v*)) and mixed with glutaraldehyde (Sigma Aldrich) at a final concentration of 0.2% (*v*/*v*) to allow polymerization. After incubation at room temperature for 4 h, mixtures were dialyzed against PBS overnight at 4 °C. Subsequently, 100 µg of HSA, MSA, BSA, as well as polymerized HSA, MSA, and BSA (pHSA, pMSA, and pBSA, respectively), were individually coupled to 200 µL of *N*-hydroxysuccimide (NHS)-activated Sepharose 4 Fast Flow (GE Healthcare, Buckinghamshire, UK; 50% slurry). PAR-deleted BNCs (ΔBNCs, BNCs lacking a large part of the pre-S region from Met-1 of the pre-S1 region to Arg-18 of the pre-S2 region [40]) were prepared by incubating BNCs with 0.2% (*w*/*w*) trypsin (Sigma Aldrich) at 37 °C for 1 h. Each albumin-conjugated resin (20 µL) was incubated with 40 µg of either BNCs or ΔBNCs in 100 µL of PBS at 37 °C for 1 h, washed with PBS 4 times, subjected to 0.1% (*w*/*v*) sodium dodecyl sulfate-12% (*w*/*v*) polyacrylamide gel electrophoresis (12% SDS-PAGE), followed by silver staining. The amounts of BNCs and ΔBNCs bound to albumin-conjugated resins were determined by densitometry using a luminescent image analyzer (LAS-4000mini; Fujifilm, Tokyo, Japan).

### 3.4. Pull-Down Assays with Peptide-Conjugated Resins

Synthetic peptides encompassing the putative PAR region (peptide 1, NH_2_-LLDPRVRGLY-COOH; peptide 2, NH_2_-TFHQALLDPRVRGLYFPA-COOH; peptide 3, NH_2_-LLDPRVRGLP-COOH; and peptide 4, NH_2_-TFHQALLDPRVRGLPFPA-COOH; underlines, mutated amino acid residues) were purchased from Scram Inc. (Tokyo, Japan). Each peptide (100 µg) was coupled to 200 μL of NHS-activated Sepharose 4 Fast Flow. Peptide-conjugated resins (40 µL; 50% slurry) were incubated with 100 µL of 1 mg/mL pHSA in PBS at 37 °C for 1 h, washed with PBS 4 times, subjected to 12% SDS-PAGE, followed by silver staining. The amounts of pHSA bound to peptide-conjugated resins were determined as described before.

### 3.5. Quartz Crystal Microbalance (QCM) Analysis

The amount of albumin bound to BNCs was determined by a QCM model Twin-Q (As One Corp., Osaka, Japan). The QCM sensor chip consisted of a 9-mm-diameter disk made from an AT-cut 27-MHz quartz crystal with gold electrodes on both sides (diameter, 2.5 mm; area, 4.9 mm^2^). A frequency change (ΔF) of 1 Hz corresponds to a weight change of 0.6 ng/cm^2^. The temperature of a measuring bath (~600 µL) was maintained at 25 °C with mixing at 600 rpm with a stirring tube. Measurements were taken in triplicate until a stable frequency (less than ± 3 Hz) was observed for >1 min. The sensor chip was treated with BNCs (2 µg/mL as protein), blocked with Block Ace (2 mg/mL; DS Pharma Biomedical, Osaka, Japan), and then reacted with each type of albumin (30 mg/mL).

### 3.6. Surface Modifications of 100-nm Polystyrene Microspheres

Monodispersed 100-nm polystyrene microspheres (Z-average, 81 nm; polydispersed index (PDI), 0.019) labeled with the fluorescent dye NYO (new yellow-orange; emission, 563 nm) and carboxyl groups were purchased from Polyscience, Inc. (Fluoresbrite Carboxylate Microspheres; Warrington, Pennsylvania, USA). Microspheres (5 mg (equivalent to 9.1 × 10^12^ particles)/mL in PBS (pH 6.0)) were incubated at room temperature for 12 min with 200 µL of 200 mg/mL sulfo-NHS (Sigma Aldrich) and 20 µL of 10 mg/mL 1-ethyl-3-(3-dimethylaminopropyl)carbodiimide (EDC; Thermo Fisher Scientific Inc., Rockford, IL, USA) in PBS (pH 6.0). The introduction of NHS residues was terminated by incubation with 6 µL of 2-mercaptoethanol (Wako Pure Chemical Industries) at room temperature for 10 min. NHS-labeled microspheres (5 mg) were purified on a Sephadex G-25 column (GE Healthcare) equilibrated with PBS (pH 7.4) and incubated at room temperature for 2 h with 1.6 mg of each HSA, MSA, lysine (Sigma Aldrich), or soybean trypsin inhibitor (STI) (Sigma Aldrich) or 1 mg of each peptide 1, 2, 3, or 4. PEGylated microspheres were also prepared by mixing lysine-labeled microspheres (5 mg) with 5 mg of PEG2000-NHS (NOF), followed by incubation at room temperature for 2 h. Average diameter (Z-average) and ζ-potential of each microsphere were measured in PBS at 25 °C using a dynamic light scattering (DLS) model Zetasizer Nano ZS (Malvern Instruments, Worcestershire, UK).

### 3.7. Phagocytosis Assay with Kupffer Cells

Kupffer cells were isolated from livers of BALB/c female mice (6–8 weeks old; CREA Japan, Tokyo, Japan) as previously described [41]. Briefly, mice were anesthetized, and the abdomen was surgically opened by a vertical incision. To prepare suspensions of nonparenchymal cells, 10 livers were treated with the gentleMACS™ Dissociator (Miltenyi Biotech, Bergisch Gladbach, Germany) in KRB buffer (pH 7.4, 154 mM NaCl, 5.6 mM KCl, 5.5 mM glucose, 20.1 mM HEPES, 25 mM NaHCO_3_, 2 mM CaCl_2_, 2 mM MgCl_2_) containing 15 mg/mL collagenase type IV and 0.07 mg/mL DNase I (Worthington Biochemical Corporation, Lakewood, NJ). Cell suspensions were passed through a 100-µm cell strainer (BD Biosciences, San Jose, CA), mixed with PEB (PBS with 0.5% (*w*/*v*) BSA and 2 mM EDTA; 25 mL, each liver), centrifuged at 20× *g* at 4 °C for 4 min to exclude hepatocytes. After centrifugation at 300× *g* at 4 °C for 10 min, pellets were suspended in 1 mL of PEB, incubated with 10 mL of ACTB [pH 7.65, 17 mM tris(hydroxymethyl)aminomethane-HCl, 0.75% (*w*/*v*) NH_4_Cl] at room temperature for hemolysis, followed by washes with 30 mL of PEB. Kupffer cells were further purified using OptiPrep™ self-forming density gradient solutions (24% (*w*/*v*), 17%, 11.5%, 8.4%, and 0% of iodixanol in PEB; Axis-Shield PoC AS, Rodeløkka, Norway), according to the manufacturer’s instruction. After centrifugation at 1.400× *g* at 4 °C for 20 min, Kupffer cells were isolated from the interphase between 11.5% and 8.4% iodixanol. After washes with PEB, cells were suspended in 1 mL of PBS and used as Kupffer cells immediately. Each NYO-labeled microsphere (50 µL; 1 × 10^11^ particles/mL) and BNCs labeled with the Fluorolink Cy3 monofunctional reactive dye (GE Healthcare) (50 µL; 1 or 10 × 10^11^ particles/mL) were opsonized by incubation with an equal volume of mouse serum (Sigma Aldrich) at 37 °C for 30 min. Kupffer cells in PBS containing 50% mouse serum were mixed with 10 µL of each opsonized microsphere, incubated at 37 °C for 30 min, and then analyzed by a flow cytometer BD FACScan Canto II (BD Biosciences), with linear amplification used for acquiring forward/side scatter and logarithmic amplification for NYO- and Cy3-derived fluorescence. Fractions of Kupffer cells were predefined by the forward scatter/side scatter dot plots derived from CD11b^+^ cells.

### 3.8. Measurement of Plasma Microsphere Concentrations

Mice were injected intravenously with 100 µL of PBS (per head) containing 100 µg of each NYO-labeled microsphere. After 10, 30, and 60 min, blood samples (200 µL) collected from eye were mixed well with 200 µL of 1 mg/mL EDTA and subsequently incubated with 100 µL of DMSO and 500 µL of chloroform. Blood samples were kept at room temperature for 3 h, with shaking to allow the solubilization of microspheres. After brief centrifugation, lower layers were isolated, and fluorescence derived from NYO-labeled microspheres in lower layers was measured using a Varioskan fluorescence microplate reader (Thermo Electron, Vantaa, Finland). Results are expressed as the mean ± SEM of five experiments.

### 3.9. Hepatocyte Uptake Assay

Hepatocytes were isolated from mouse livers as previously described [41]. Briefly, mice were anesthetized, and the abdomen was surgically opened by a vertical incision. Livers were perfused via the portal vein with Gey’s balanced salt solution (GBSS) without Ca^2+^ (pH 7.4, 0.14 M NaCl, 5 mM KCl, 0.3 mM MgSO_4_, 1 mM NaH_2_PO_4_, 3 mM NaHCO_3_, 0.2 mM KH_2_PO_4_, 1 mM MgCl_2_, 5.5 mM glucose) at a flow rate of 5 mL/min for 10 min, followed by perfusion with GBSS with Ca^2+^ (pH 7.4, 0.14 M NaCl, 5 mM KCl, 0.3 mM MgSO_4_, 1 mM NaH_2_PO_4_, 1.5 mM CaCl_2_, 3 mM NaHCO_3_, 0.2 mM KH_2_PO_4_, 1 mM MgCl_2_, 5.5 mM glucose) containing 0.2 mg/mL collagenase type IV, 2% BSA, and 0.1 mM CaCl_2_ for 10–15 min. Next, livers were excised and minced, and cell suspensions were passed through a 100-µm cell strainer. After centrifugation at 50× *g* at 4 °C for 3 min, hepatocytes separated from nonparenchymal cells were suspended in 1 mL of PBS and used immediately. Each NYO-labeled microsphere (50 µL; 1 or 2 × 10^11^ particles/mL) and Cy3-labeled BNCs (50 µL; 2 or 20 × 10^11^ particles/mL) were opsonized by incubation with an equal volume of mouse serum at 37 °C for 30 min. Hepatocytes in PBS (100 µL; 5.0 × 10^5^ cells/mL) were mixed with 10 µL of each opsonized microsphere, incubated at 37 °C for 30 min, and then analyzed by a flow cytometer BD FACScan Canto II, with linear amplification for forward/side scatter and logarithmic amplification for NYO- and Cy3-derived fluorescence.

### 3.10. Statistics

Statistical significance was evaluated by using Student’s *t*-test

## 4. Conclusions

Through the surface modification with HBV-derived PAR peptides, we demonstrate that NPs were able to escape from the uptake by Kupffer cells efficiently in vitro and therefore show a RES evasion in vivo. We also show that hepatocytes incorporated fewer NPs displaying PAR peptides than albumin-modified NPs, both in vitro and in vivo. These results strongly suggest that the modification with PAR peptides is an alternative strategy for improving the pharmacodynamics and pharmacokinetics of forthcoming nanomedicines. Moreover, BNC has been considered as an ideal scaffold for oriented immobilization of PAR peptides and human hepatocyte-recognizing domains [42], both of which might concurrently contribute to high human liver-specific infectivity and efficient RES evasion activity. Recently, we have succeeded in retargeting BNCs to non-human hepatic cells by replacing the human hepatocyte-recognizing domain with other biorecognition molecules, including antibodies [43] and cytokines [13]. In these cases, the retargeting of BNCs is necessary to retain PAR peptides for efficient RES evasion.

## Figures and Tables

**Table 1:** Sizes and surface charges of BNCs, LPs, and BNC-LP complexes.

Sample	Z-Average (nm)	PDI	ζ-Potential (mV)
LP	151	0.318	–74.8
BNC	70.8	0.186	–15.6
BNC-LP	157	0.356	–43.5

**Table 2:** QCM analysis of interactions between albumins and BNCs.

Albumin	Albumin/L Protein (mol ratio) N = 4	Albumin/BNC (mol ratio) * N = 3
HSA	0	0
MSA	0	0
pHSA	0.053 ± 0.017	5.820 ± 1.838
pMSA	0.011 ± 0.007	1.174 ± 0.741

Values ± SD. * Based on the calculation that each BNC particle has about 110 L proteins [29].

**Table 3:** Sizes and surface charges of surface-modified microspheres.

Microspheres	Z-Average (nm)	PDI	ζ-Potential (mV)
Naked	81.0	0.019	–53.6
PEG	95.2	0.186	–45.9
HSA	120	0.120	–31.3
MSA	102	0.019	–29.3
Peptide 2	78.0	0.010	–54.7
STI	106	0.127	–25.5

## Data Availability

All data generated or analyzed during this study are included in this published article. Further datasets used and/or analyzed during the study are available from the corresponding author on reasonable request.

## Abbreviations

BNC: bio-nanocapsule; DDS, drug delivery system; ΔBNC, PAR-deleted BNC; FcRn, neonatal Fc receptor; HBV, hepatitis B virus; HBsAg, HBV surface antigen; HSA, human serum albumin; LP, liposome; MSA, mouse serum albumin; NHS, *N*-hydroxysuccimide; NP, nanoparticle; PAR, polymerized human serum albumin receptor; PDI, polydispersity index; PEG, polyethylene glycol; pHSA, polymerized human serum albumin; pMSA, polymerized mouse serum albumin; QCM, quartz crystal microbalance; RES, reticuloendothelial system; Rh, Lissamine rhodamine B; STI, soybean trypsin inhibitor.
